# GAS5 rs2067079 and miR-137 rs1625579 functional SNPs and risk of chronic hepatitis B virus infection among Egyptian patients

**DOI:** 10.1038/s41598-021-99345-2

**Published:** 2021-10-08

**Authors:** Rania H. Mahmoud, Enas Mamdouh Hefzy, Olfat G. Shaker, Tarek I. Ahmed, Noha K. Abdelghaffar, Essam A. Hassan, Amal A. Ibrahim, Doaa Y. Ali, Mohamed M. Mohamed, Omayma O. Abdelaleem

**Affiliations:** 1 Department of Medical Biochemistry and Molecular Biology, Faculty of Medicine, Fayoum University, Fayoum, Egypt; 2 Department of Medical Microbiology and Immunology, Faculty of Medicine, Fayoum University, Fayoum, 63514 Egypt; 3 Department of Medical Biochemistry and Molecular Biology, Faculty of Medicine, Cairo University, Cairo, Egypt; 4 Department of Internal Medicine, Faculty of Medicine, Fayoum University, Fayoum, Egypt; 5 Department of Clinical Pathology, Faculty of Medicine, Fayoum University, Fayoum, Egypt; 6 Department of Tropical Medicine, Faculty of Medicine, Fayoum University, Fayoum, Egypt; 7 Department of Internal Medicine, Faculty of Medicine, Cairo University, Cairo, Egypt

**Keywords:** Biochemistry, Genetics, Microbiology, Diseases, Medical research, Risk factors

## Abstract

Hepatitis B virus (HBV) infection is a significant health issue worldwide.. We attempted to fulfill the molecular mechanisms of epigenetic and genetic factors associated with chronic HBV (CHBV). Expression levels of the lncRNA growth arrest-specific 5 (GAS5) and miR-137 and their corresponding SNPs, rs2067079 (C/T) and rs1625579 (G/T) were analyzed in 117 CHBV patients and 120 controls to investigate the probable association between these biomarkers and CHBV pathogenesis in the Egyptian population. Serum expression levels of GAS5 and miR-137 were significantly down-regulated in cases vs controls. Regarding GAS5 (rs2067079), the mutant TT genotype showed an increased risk of CHBV (*p* < 0.001), while the dominant CC was a protective factor (*p* = 0.004). Regarding miR-137 rs1625579, the mutant genotype TT was reported as a risk factor for CHBV (*p* < 0.001) and the normal GG genotype was a protective factor, p < 0.001. The serum GAS5 was significantly higher in the mutant TT genotype of GAS5 SNP as compared to the other genotypes (*p* = 0.007). Concerning miR-137 rs1625579, the mutant TT genotype was significantly associated with a lower serum expression level of miR-137 (*p* = 0.018). We revealed the dysregulated expression levels of GAS5 and miR-137 linked to their functioning SNPs were associated with CHBV risk and might act as potential therapeutic targets.

## Introduction

Hepatitis B virus (HBV), a double-stranded DNA virus, is a member of the Hepadnavirus family. HBV infection leads to hepatic acute and chronic diseases. HBV infection causes the development of various liver diseases including hepatocellular carcinoma (HCC), cirrhosis, acute and chronic hepatitis^1^. Less than 5% of infected healthy adults will develop chronic HBV infection^2^. It was reported that about 54.4% of HCC cases are caused by HBV infection^3^. According to World’s Health Organization (WHO), more than 257 million persons globally are living with HBV, leading to nearly one million deaths annually^4^. The frequency of hepatitis B surface antigen (HBsAg) was expected to be 0.8% among Egyptians aged from 1 to 59 years^5^.

Noncoding RNAs (ncRNAs) are defined as RNAs possessing the little capability of protein‐coding. They function as promising regulators of epigenetic, transcriptional, and post-transcriptional gene expression. Noncoding RNAs are divided into long ncRNAs (lncRNAs), small interfering RNAs (siRNAs), microRNAs (miRNAs), and transfer RNAs (tRNAs). LncRNAs are RNA molecules whose length exceeds 200 nucleotides and not translated into proteins. However, accurate classification and identification of lncRNAs are still not available^6^. LncRNAs are concerned with several biological processes, including epigenetic regulation of gene expression, apoptosis, cell-cycle control, as well as in the development, differentiation, and senescence of cells^7^.

Studies reported that the lncRNA growth arrest-specific 5 (GAS5) is needed for arrest of normal growth, and slowing of the cell cycle^8^. It also has diverse functions in regulating gene expression, induction of cell apoptosis, suppression of tumorigenesis^9^, and inhibition of T-cell proliferation^10^. The role of GAS5 in benign liver diseases has been determined in hepatitis C virus (HCV) replication and liver fibrosis. GAS5 binds the multifunctional NS3 protein, which has a serine protease activity and inhibits HCV replication^11^. Though, its function in HBV infection remains mostly unidentified. GAS5 genomic variants were also associated with multiple cancers and autoimmune disease risk^9^.

MiR-137, which is the target miRNA of GAS5, was reported to have a suppressive role in HCC^12^. MiR-137 expression level is lowered in HCC. Moreover, miR-137 down-regulation was significantly linked to vein invasion and remote metastasis^13^. However, little is known about the role of miR-137 in chronic liver diseases.

Numerous researches have suggested that gene polymorphisms of non-coding RNAs are associated with chronic hepatitis B virus infection (CHBV) risk^14,15^. However, the association between GAS5 rs2067079 (C/T) and miR-137 rs1625579 (G/T) with chronic HBV (CHBV) has not been studied yet.

We hypothesized that the variations in the serum expression levels of lncRNA GAS5 and MiR-137 which can be affected by their relevant SNPs; GAS5 rs2067079 (C/T) and miR-137 rs1625579 (G/T), respectively, can raise the risk of development of CHBV infection. This hypothesis motivated us to investigate the association between these SNPs with CHBV infection as well as its effect on the expression of serum GAS5 and miR-137. Moreover, the possible association of rs2067079 and rs1625579 SNPs with various clinical and laboratory characteristics of these patients was assessed.

## Results

### Demographic and clinical characterization of the study groups

**Figure 1:**
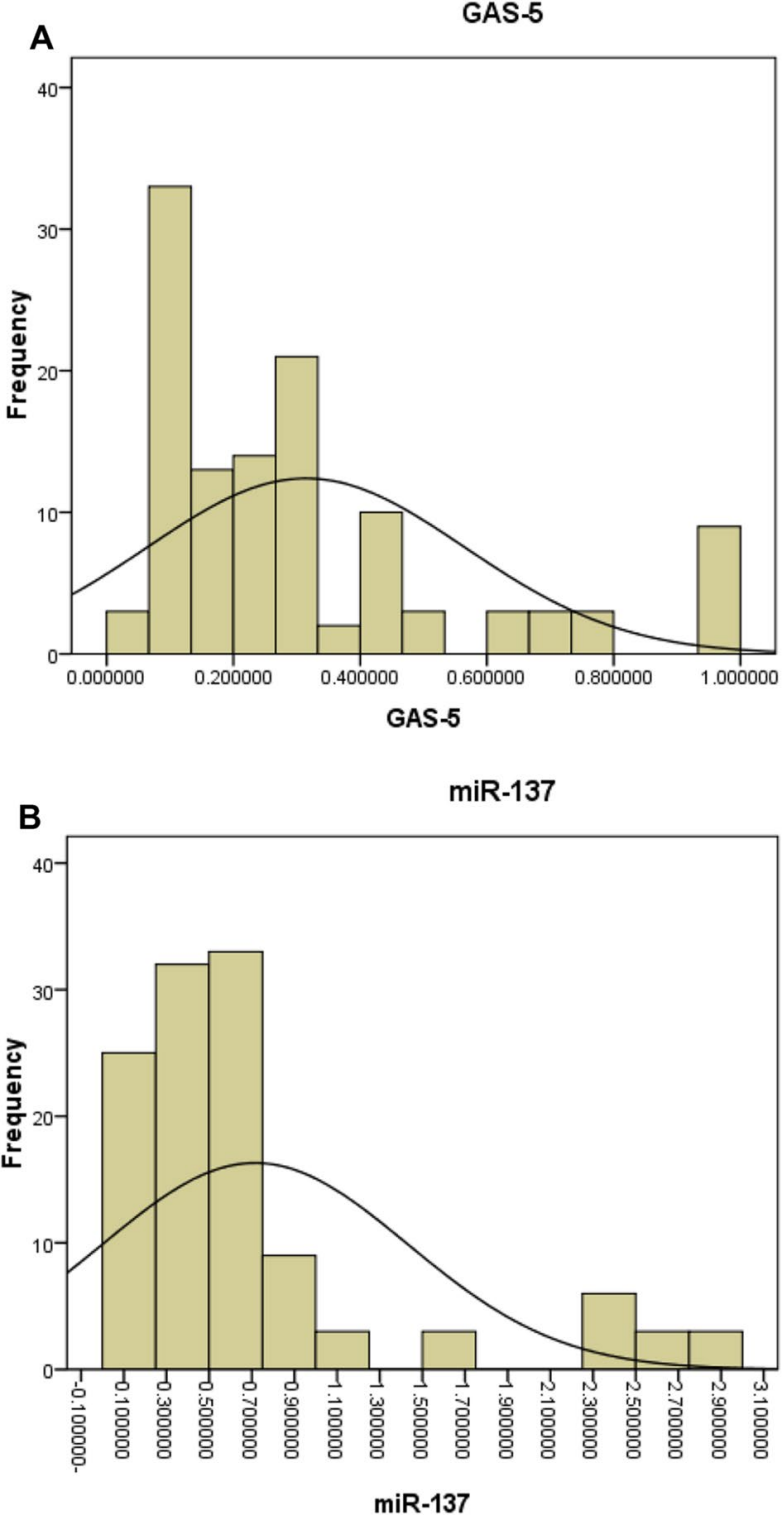
A frequency curve represents the serum expression levels (expressed as fold changes) of: **(a)** lncRNA growth arrest-specific 5 (GAS5), **(b)** miR-137; among chronic HBV cases Expression level among control subjects was considered as 1.

This case–control study included 117 patients with chronic HBV infection and 120 controls that had previous HBV infection and completely recovered from infection. Among the patients, the male/female ratio was 3.88 and it was 3.81 for control. No significant difference was observed between cases and controls regarding age and sex (*p* = 0.206 and 0.951, respectively) (Table 1). Table 1 also demonstrates the clinical and laboratory data of the patients recruited in the study. In CHBV patients, the serum expression level of GAS5 and miR-137 was significantly down-regulated in cases vs controls, *p* < 0.001 for each (Table 1 and Fig. 1).

**Table 1:** Demographic, clinical and laboratory of study subjects.

	Cases (N = 117)	Control (n = 120)	P value
**Sex (N %)**			
Male Female	93 (79.5%) 24 (20.5%)	95 (79.2%) 25 (20.8%)	0.951
**HBeAg**			
Positive Negative	25 (21.4%) 92 (78.6%)		
**HBV viral load**			
BDL Low load High load	40 (34.2%) 50 (42.7%) 27 (23.1%)		

	Mean ± SEM		
Age (years)	40.59 ± 1.26	42.62 ± 1.00	0.206
BMI (kg/m^2^)	27.07 ± 0.86	28.8 ± 0.19	0.409
Hb (g/dl)	13.27 ± 0.22	13.2 ± 0.11	0.240
Creatinine (mg/dl)	0.86 ± .022	0.8 ± 0.03	0.057
FBS (mg/dl)	126.59 ± 8.88	120.81 ± 2.8	0.327
ALT (U/L)	43.83 ± 1.08	39.3 ± 0.69	0.003
AST (U/L)	38.36 ± 0.88	32.7 ± 0.58	< 0.001*
Albumin (g/dl)	4.11 ± 0.08	4.61 ± 0.04	< 0.001*
Total bilirubin (mg/dl)	0.93 ± 0.16	0.76 ± 0.02	0.262
Direct bilirubin (mg/dl)	0.51 ± 0.18	0.55 ± 0.02	0.829
TLC (1/mm^3^)	4626.57 ± 350.8	8500.4 ± 137.01	< 0.001*
PLT (1/mm^3^)	1.7 × 10^5^ ± 13,290	34.2*10^4^ ± 114.14	< 0.001*
INR	1.09 ± 0.01	0.81 ± 0.02	< 0.001*
IQR	0.28 ± 0.02		
Stiffness (kPa)	12.17 ± 1.7		
Interquartile range/liver stiffness ratio, mean (SD)	0.034 ± 0.003		
AFP (µg/L)	5.29 ± 0.395		
GAS-5	0.32 ± 0.02	1 ± 0	< 0.001*
miR-137	0.72 ± 0.07	1 ± 0	< 0.001*

Data are expressed as mean ± SEM (standard error of mean); expression values of GAS5 and miR-137 in control group were set as 1.

*BMI* body mass index, *GAS-5* lncRNA growth arrest-specific 5, *miR-137* microRNA 137, *AFP* alpha fetoprotein, *FBS* fasting blood sugar, *IQR* interquartile range, *Hb* hemoglobin, *TLC* total leucocytic count, *PLT* platelet count, *INR* the international normalized ratio, *kPa* kilopascals, *ALT* alanine aminotransferase, *AST* aspartate aminotransferase, *BDL* below detection level (below 20 copies/ml), *low load* viral load 20–10^4^ copies/ml, *high load* viral load > 10^4^ copies/ml.

*Significant.

### Diagnostic performance of GAS5 and miR-137 in CHBV

**Figure 2:**
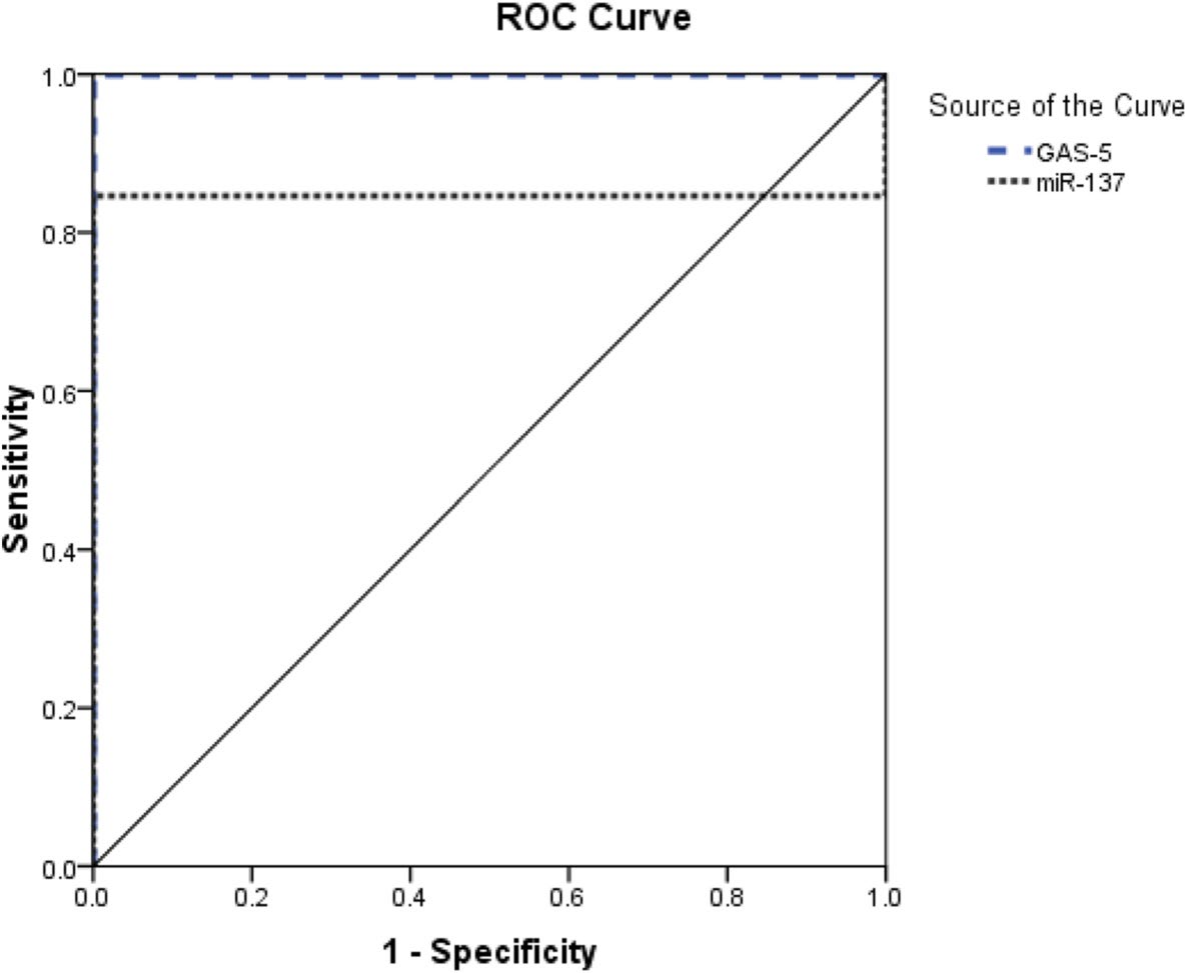
ROC curve demonstrates the role of lncRNA growth arrest-specific 5 (GAS5) and microRNA 137 (miR-137) in diagnosis of chronic viral hepatitis B cases.

Receiver-operating-characteristic (ROC) curve analysis revealed that GAS5 can distinguish CHBV patients from controls (AUC = 1, 95% CI (1 − 1), *p* < 0.001) with a sensitivity of 100% and a specificity of 100% at a cutoff > 0.98 (fold). Serum miR-137 also distinguished CHBV patients from controls (AUC = 0.846, 95% CI (0.78–0.91), *p* < 0.001) with a sensitivity and specificity of 84.6% and 100%, respectively, at a cutoff point 0.96 (fold) (Fig. 2).

### Association of GAS5 rs2067079 (C/T) and miR-137 rs1625579 (G/T) with the risk of CHBV

The strength of the association between the SNPs and risk of CHBV was assessed by the ORs with the corresponding 95% CI for the following genetic models: (1) the codominant model (C/C vs C/T vs T/T for rs2067079 and G/G vs G/T vs T/T for rs1625579), (2) the dominant genetic model: (CC vs T/T + C/T) (C is the major allele and T is the minor allele for rs2067079) and (G/G vs T/T + G/T) (G is the major allele and T is the minor allele for rs1625579), (3) the recessive genetic model (T/T vs. C/T + C/C and T/T vs. G/T + G/G for rs2067079 and rs1625579, respectively), (4) the over-dominant model (CT vs CC + TT and GT vs GG + TT for rs2067079 and rs1625579, respectively) and (5) C allele vs the T allele and G allele vs T allele analysis for rs2067079 and rs1625579, respectively) (Table 2).

**Table 2:** Association of GAS5 rs2067079 (C/T) and miR-137 rs1625579 (G/T) with the risk of chronic hepatitis B viral infection.

		Cases (N%) (N = 117)	Control (N%) (n = 120)	OR (95%CI)	P value
**rs2067079 (C/T)**					
**Codominant model**	CC	39 (33.3%)	62 (51.7%)		0.001*
	CT	57 (48.7%)	54 (45.0%)	8.34 (2.7–26.14)	< 0.001*
	TT	21 (17.9%)	4 (3.3%)	4.9 (1.6–15.4)	0.005*
**Recessive model (TT vs CT + CC)**	TT	21 (17.9%)	4 (3.3%)	6.34 (2.1–19.11)	< 0.001*
	CT&CC	96 (82.1%)	116 (96.7%)		
**Overdominant model (CT vs CC + TT)**	CT	57 (48.7%)	54 (45%)	1.16 (0.7–1.94)	0.566
	CC&TT	60 (51.3%)	66 (55%)		
**Dominant model (CC vs CT + TT)**	CC	39 (33.3%)	62 (51.7%)	0.47 (0.28–0.79)	0.004*
	CT&TT	78 (66.7%)	58 (48.3%)		
**Allele**	C	135 (57.7%)	178 (74.2%)	2.1 (1.4–3.11)	0.001*
	T	99 (42.3%)	62 (25.8%)		
**rs1625579 (G/T)**					
**Codominant model**	GG	12 (10.3%)	45 (37.5%)		< 0.001*
	GT	27 (23.1%)	64 (53.3%)	1.58 (0.72–3.44)	0.247
	TT	78 (66.7%)	11 (9.2%)	26.59 (10.85–65.2)	< 0.001*
**Dominant model (GG vs GT + TT)**	GG	12 (10.3%)	45 (37.5%)	0.19 (0.09–0.38)	< 0.001*
	GT&TT	105 (89.7%)	75 (62.5%)		
**Overdominant model (GT vs GG + TT)**	GT	27 (23.1%)	64 (53.3%)	0.26 (0.15–0.46))	< 0.001*
	GG&TT	90 (76.9%)	56 (46.7%)		
**Recessive model (TT vs GG + GT)**	TT	78 (66.7%)	11 (9.2%)	19.8 (9.56–41.1)	< 0.001*
	GG&GT	39 (33.3%)	109 (90.8%)		
**Allele**	G	51 (21.8%)	154 (66.2%)	2 (1.33–3.01)	< 0.001*
	T	183 (78.2%)	86 (33.8%)		

For rs2067079 (C/T), C is the dominant allele, for rs1625579 (G/T), G is the dominant allele.

*GAS-5* lncRNA growth arrest-specific 5, *miR-137* microRNA 137, *OR* odds ratio, *CI* confidence interval.

*Significant.

For both SNPs, the minor allele frequency (MAF) in the controls was 25.8% for rs2067079 and 33.8% for rs1625579, which was slightly higher than the global MAF (T = 19% for rs2067079 and G = 20% for rs1625579) as reported in Ensembl GRCh37 release^16^.

The genotypes distribution of rs2067079 and rs1625579 in the control did not significantly deviate from what was expected by the Hardy–Weinberg equilibrium (HWE) (*p* = 0.056 and 0.08 respectively).

### Association of GAS5 rs2067079 (C/T) with the risk of CHBV

The genotype and allele frequencies for rs2067079 in CHBV patients and control subjects are shown in Table 2. Regarding the rs2067079, the recessive T allele predominated among cases (42.3% vs 25.8% in controls) and the dominant C allele predominated among controls (74.2% vs 57.7% in cases). In the co-dominant model (CC vs CT vs TT), the distribution of the rs2067079 genotypes was significantly different between CHBV patients and control subjects (CT genotype vs CC genotype, *p* < 0.001; and TT genotype vs CC genotype, *p* = 0.005). The CT and TT genotypes represented an increased risk of CHBV for about 8 and 5 folds respectively. According to the recessive model (TT vs CC + CT), the TT genotype showed a 6.3-fold increased risk of CHBV, [OR (95%CI): 6.34 (2.1–19.11), *p* < 0.001]. In the dominant model (CC vs CT + TT), the CC genotype was defined as a protective factor against CHBV risk, [OR (95%CI): 0.468 (0.277–0.791), *p* = 0.004] (Table 2).

### Association of miR-137 rs1625579 (G/T) with the risk of CHBV

As reported in Table 2, regarding rs1625579, the mutant TT genotype and the mutant T allele were predominantly found in CHBV cases vs controls (66.7% vs 9.2% and 78.2% vs 33.8% for TT genotype and T allele, respectively). The mutant T allele had a double-fold risk of CHBV vs the normal G allele [OR (95%CI), 2 (1.33–3.01), *p* < 0.001]. In the codominant model (TT vs GT vs GG), the frequency of the rs1625579 genotypes was significantly different between CHBV patients and control subjects. The dominant GG genotype predominates among cases vs controls (10.3% vs 37.5%, *p* < 0.001) and the mutant TT genotype predominates among controls vs cases (66.7% vs 9.2% *p* < 0.001).

The mutant TT genotype was a significant risk factor for CHBV, [OR (95%CI), 26.59 (10.85–65.2) *p* < 0.001]. The normal GG genotype was a significant protective factor against CHBV in the dominant model (GG vs GT + TT), [OR (95%CI) 0.19 (0.094–0.3840, *p* < 0.001]. The mutant genotype TT was found as a significant risk factor for CHBV as regards the recessive model (TT vs GG + GT), [OR (95%CI), 19.8 (9.56–41.1), *p* < 0.001] (Table 2).

### Association of serum GAS5 and miR-137 levels with rs2067079 and rs1625579 in CHBV patients

**Figure 3:**
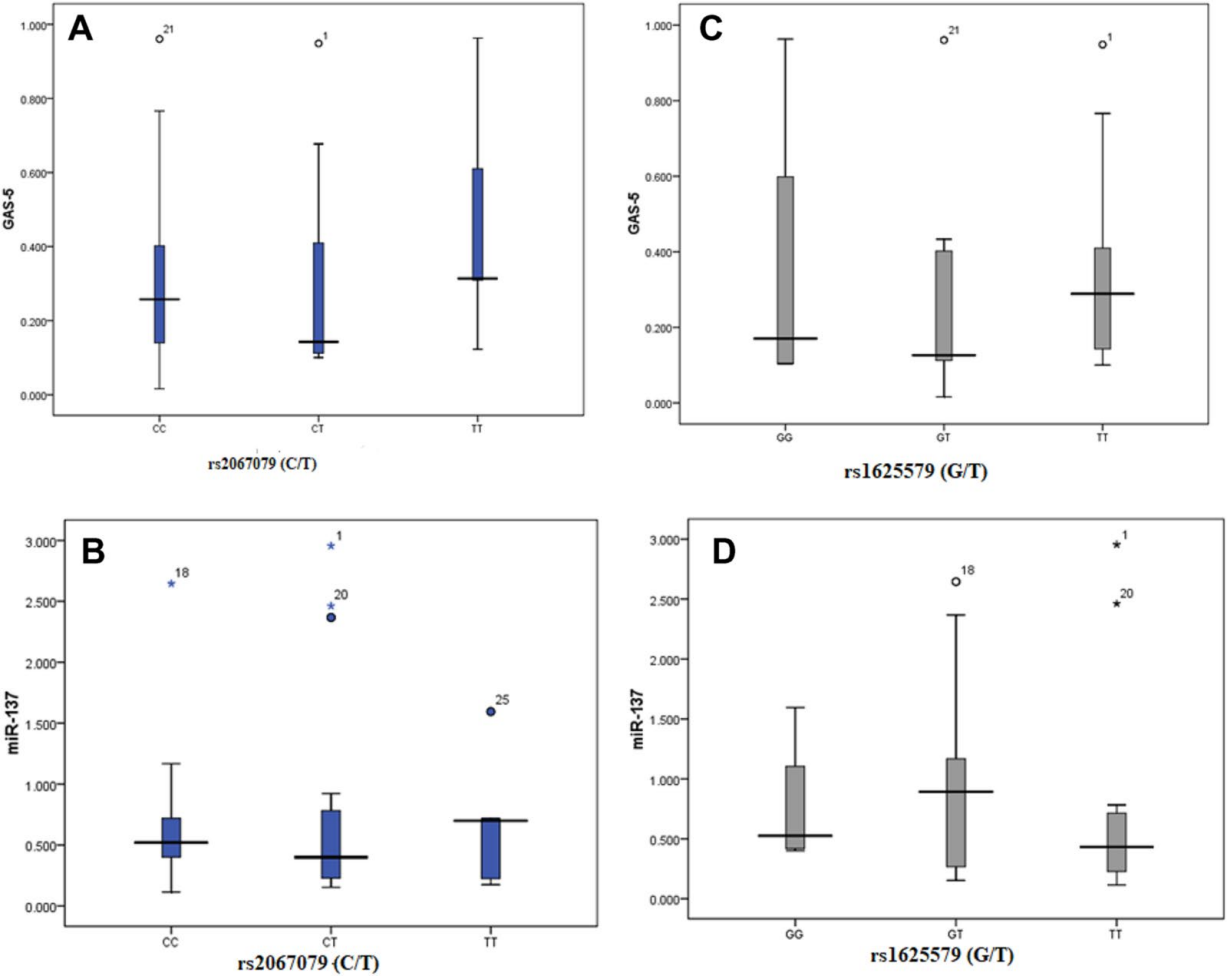
Relation of rs2067079 and rs1625579 genotypes and serum expression levels of GAS5 and miR-137 in chronic viral hepatitis B cases. The box displays the 25% − 75% percentiles; the median is represented by the line inside the box and the upper and lower lines represent the 10% to 90% percentiles of the serum level (fold change) **(a)** GAS5 in different rs2067079 genotypes and **(b)** miR-137 in different rs2067079 genotypes; CC (N = 39), CT (N = 57), TT (N = 21) and serum **(c)** miR-137 in different rs1625579 genotypes **(d)** and GAS5 in different rs1625579 genotypes; GG (n = 12), GT (n = 27), TT (n = 78).

We evaluated serum expression levels of GAS5 and miR-137 in CHBV patients having the different SNP genotypes to interpret the role of rs2067079 and rs1625579 in this disease (Fig. 3). By studying the effect of rs2067079, we found that the serum GAS5 expression level was significantly higher in the mutant TT genotype carriers vs those with the CT or CC genotypes (*p* = 0.007) (Fig. 3a). However, this SNP did not affect the expression level of miR-137 (*p* = 0.056) (Fig. 3b).

Regarding the rs1625579, we did not find a significant difference in the serum expression of GAS5 among CHBV patients with different rs1625579 genotypes (*p* = 0.489) (Fig. 3c). In contrast, serum miR-137 expression level was significantly down-regulated with the mutant TT genotype versus GT and GG genotypes among CHBV patients (*p* = 0.018) (Fig. 3d).

### Effect of GAS5 rs2067079 (C/T) and miR-137 rs1625579 (G/T) on demographic, clinical, and laboratory variables of chronic viral hepatitis B patients

By studying the effect of GAS5 rs2067079 (C/T) on demographic, clinical, and laboratory variables of CVHB B patients (Table 3), the recessive TT genotype was significantly associated with low body mass index (BMI), *p* = 0.001, and with high GAS5 serum expression level, *p* = 0.007. The CT genotype was predominantly associated with males *p* = 0.033.

**Table 3:** Effect of GAS5 rs2067079 (C/T) on demographic, clinical and laboratory variables of chronic viral hepatitis B patients.

	GAS5 rs2067079 (C/T) genotypes			**P value**
	CC (N = 39)	CT (N = 57)	TT (N = 21)	
Sex (male/female) (N)	12/27	6/51	6/15	0.033*

	Mean ± SD			
Age (years)	43.15 ± 15.46	39.00 ± 13.08	40.14 ± 10.81	0.237
BMI (kg/m^2)^	30.75 ± 0.82	26.50 ± 3.66	22.00 ± 0.0	0.001*
Hb (g/dl)	12.700 ± 2.2396	13.561 ± 2.1370	13.533 ± 2.9350	0.201
Creatinine (mg/dl)	0.9 ± 0.24	0.83 ± 0.20	0.87 ± 0.25	0.373
FBS (mg/dl)	134.00 ± 45.837	139.87 ± 85.437	92.25 ± 7.545	0.094
ALT (U/L)	45.15 ± 8.19	43.56 ± 11.62	42.00 ± 15.04	0.575
AST (U/L)	36.54 ± 9.84	39.69 ± 6.63	38.71 ± 12.34	0.279
Albumin (g/dl)	3.86 ± 1.12	4.18 ± 0.47	4.35 ± 0.44	0.05
INR	1.11 ± 0.12	1.09 ± 0.14	1.03 ± 0.052	0.108
Stiffness	15.18 ± 22.0	12.15 ± 18.70	6.64 ± 2.84	0.55

	Median (IQR)			
TLC (1/mm^3^)	4150 (281–9325)	5350 (1110–7400)	4950 (4600–5600)	0.944
PLT (1/mm^3^)	1.24 × 10^5^ (5966–2.95 × 10^5)^	2.31 × 10^5^ (3.1 × 10^4^–2.91 × 10^5)^	1.9 × 10^5^ (1.4 × 10^4^–2.1 × 10^5)^	0.571
Total bilirubin (mg/dl)	0.64 (0.4–0.9)	0.76 (0.58–0.9)	0.6 (0.4–0.65)	0.130
Direct bilirubin (mg/dl)	0.2 (0.1–0.3)	0.19 (0.1–0.3)	0.15 (0.1–0.15)	0.268
IQR	0.28 (0.16–0.52)	0.23 (0.11–0.51)	0.18 (0.10–0.23)	0.084
AFP (µg/L)	6.0 (10–9)	6.0 (3–9)	6 (2–9)	0.943
GAS-5	0.26 (0.10–0.40)	0.14 (0.11–0.41)	0.31 (0.31–0.61)	0.007*
miR-137	0.52 (0.40–0.72)	0.40 (0.23–0.78)	0.70 (0.23–0.72)	0.489

*BMI* body mass index, *GAS-5* lncRNA growth arrest-specific 5, *miR-137* microRNA 137, *AFP* alpha fetoprotein, *FBS* fasting blood sugar, *IQR* interquartile range, *SD* standard deviation, *Hb* hemoglobin, *TLC* total leucocytic count, *PLT* platelet count, *INR* the international normalized ratio, *kPa* kilopascals, *ALT* alanine aminotransferase, *AST* aspartate aminotransferase.

*Significant.

On the other hand, as regards the miR-137 rs1625579 (G/T) effect on patients’ characteristics (Table 4), the mutant TT genotype was significantly associated with young age (*p* = 0.033), high levels of total and direct bilirubin (*p* = 0.011 and < 0.001, respectively), and lower serum expression level of miR-137 (*p* = 0.018). The GT genotype was associated with high fasting blood sugar (FBS) (*p* = 0.011).

**Table 4:** Effect of miR-137 rs1625579 (G/T) on demographic, clinical and laboratory variables of chronic viral hepatitis B patients.

	miR-137 rs1625579 (G/T) genotypes			**P value**
	GG (N = 12)	GT (N = 27)	TT (N = 78)	
Sex (male/female) (N)	3/9	6/21	15/63	0.871

	Mean ± SD			
Age (years)	43.15 ± 15.46	44.22 ± 12.67	38.35 ± 12.79	0.033*
BMI (kg/m^2^)	30.00 ± 0.00	27.25 ± 4.66	26.25 ± 3.93	0.355
Hb (g/dl)	13.00 ± 3.031	13.63 ± 2.20	13.19 ± 2.30	0.684
Creatinine (mg/dl)	0.98 ± 0.17	0.91 ± 0.32	0.84 ± 0.19	.098
FBS (mg/dl)	86.00 ± 4.38	152.50 ± 38.89	128.85 ± 68.71	0.011*
ALT (U/L)	34.25 ± 11.96	36.33 ± 20.52	39.73 ± 23.1	**0.614**
AST (U/L)	48.50 ± 31.04	39.12 ± 6.75	37.42 ± 9.98	0.186
Albumin (g/dl)	4.45 ± 0.49	3.74 ± 1.26	4.20 ± 0.46	0.251
INR	1.08 ± 0.059	1.07 ± 0.14	1.10 ± 0.15	0.183

	Median (IQR)			
TLC (1/mm^3^)	5600 (4.9–9860)	3655 (5.3–7775)	4600 (1110–6580)	0.944
PLT (1/mm^3^)	1.93 × 10^5^ (288–2.24 × 10^5)^	1.06 × 10^5^ (215.5–2.9 × 10^5)^	2.17 × 10^5^ (3.1 × 10^4^–3.25 × 10^5)^	0.571
Total bilirubin (mg/dl)	0.4 (0.31–0.6)	0.64 (0.4–0.8)	0.7 (0.5–0.9)	0.011*
Direct bilirubin (mg/dl)	0.1 (0.06–0.1)	0.16 (0.1–0.2)	0.19 (0.12–0.3)	< 0.001*
Stiffness	6 (5.15–7)	6.1 (5.8–7.8)	5.3 (4.5–8.1)	0.315
IQR	0.17 (0.09–0.24)	0.49 (0.05–0.59)	0.24 (0.13–0.29)	0.217
AFP (µg/L)	6.0 (10–9)	6.0 (3–9)	6 (2–9)	0.943
GAS-5	0.17 (0.10–0.78)	0.13 (0.11–0.40)	0.29 (0.14–0.41)	0.06
miR-137	0.53 (0.41–1.35)	0.89 (0.27–1.17)	0.43 (0.23–0.72)	0.018*

*BMI* body mass index, *GAS-5* lncRNA growth arrest-specific 5, *miR-137* microRNA 137, *AFP* alpha fetoprotein, *FBS* fasting blood sugar, *IQR* interquartile range, *SD* standard deviation, *Hb* hemoglobin, *TLC* total leucocytic count, *PLT* platelet count, *INR* the international normalized ratio, *kPa* kilopascals, *ALT* alanine aminotransferase, *AST* aspartate aminotransferase.

*Significant.

### Correlation study of GAS5 and miR-137 serum expression levels with clinical and laboratory characteristics of CHBV cases

It was noted that both GAS5 and miR-137 serum expression levels had a significantly positive association with age (*p* < 0.0001 & *p* = 0.046, respectively), serum creatinine (*p* = 0.003 & *p* = 0.013, respectively), direct bilirubin (*p* < 0.0001, for each), and total bilirubin (*p* < 0.0001, for each), and significant negative association with albumin level (*p* = 0.033 and *p* < 0.0001, respectively), and degree of liver stiffness (*p* = 0.038 & *p* = 0.049, respectively). GAS5 serum expression level had a significantly positive association with ALT (*p* = 0.015) and a significantly negative association with FBS (*p* < 0.0001), IQR (*p* = 0.023), and AFP (*p* = 0.006). GAS5 and miR-137 had a significant positive association (*p* = 0.012) (Table 5).

**Table 5:** Correlation study of GAS5 and miR-137 serum expression levels with clinical and laboratory characteristics of CHBV cases.

		GAS-5	miR-137
**miR-137**	*r*	0.233	1
	P value	0.012*	
**Age (years)**	*r*	0.360	0.185
	P value	< 0.0001*	0.046*
**Creatinine (mg/dl)**	*r*	0.292	0.241
	P value	0.003*	0.013*
**FBS (mg/dl)**	*r*	−0.521	−0.084
	P value	< 0.0001*	0.556
**ALT (U/L)**	*r*	0.233	0.171
	P value	0.015*	0.076
**Total bilirubin (mg/dl)**	*r*	0.429	0.513
	P value	< 0.0001*	< 0.0001*
**Direct bilirubin (mg/dl)**	*r*	0.429	0.561
	P value	< 0.0001*	< 0.0001*
**Albumin (g/dl)**	*r*	−0.212	−0.624
	P value	0.033*	< 0.0001*
**Stiffness**	*r*	−0.192	−0.182
	P value	0.038*	0.049*
**IQR**	*r*	−0.274	0.190
	P value	0.023*	0.118
**AFP (µg/L)**	*r*	−0.512	−0.113
	P value	0.006*	0.574

*GAS-5* lncRNA growth arrest-specific 5, *miR-137* microRNA 137, *AFP* alpha fetoprotein, *FBS* fasting blood sugar, *IQR* interquartile range, *ALT* alanine aminotransferase.

*Significant.

No association was found between GAS-5 serum expression level and presence of HBeAg (*p* = 0.736) or HBV viral load in the serum of CHBV cases (*p* = 0.263). Similarly, miR-137 had no association with HBeAg (*p* = 0.709), but it was significantly high with very low HBV viral load (less than 20 copies/ml) among CHBV cases (*p* = 0.035) (Table 6).

**Table 6:** Relation between HBeAg and HBV viral load and lncRNA growth arrest-specific 5 (GAS5) and miR-137 serum expression levels among chronic HBV cases.

		GAS-5 (fold change)	miR-137 (fold change)
**HBeAg**	Positive (N = 25)	0.32 ± 0.03	0.73 ± 0.07
	Negative (N = 92)	0.3 ± 0.06	0.67 ± 0.16
	P-value	0.736	0.709
**HBV viral load**	BDL (N = 40)	0.30 ± 0.04	0.95 ± 0.12
	Low load (N = 50)	0.35 ± 0.04	0.60 ± 0.09
	High load (N = 27)	0.26 ± 0.05	0.58 ± 0.14
	P-value	0.263	0.035*

*BDL* below detection level (below 20 copies/ml), *low load* viral load 20–10^4^ copies/ml, *high load* viral load > 10^4^ copies/ml.

*Significant *p* value.

### Logistic regression analysis

In our study, in the multivariate analysis, the rs2067079 mutant TT genotype (*p* = 0.033) and the rs1625579 mutant TT genotype (*p* < 0.0001) were revealed to be significant positive independent predictors of CHBV risk (Table 7).

**Table 7:** Logistic regression analysis.

Predictors	B	P value	Odds	95.0% CI	
				Lower	Upper
Age	− 0.013	0.355	0.987	0.960	1.015
Sex	0.060	0.889	1.062	0.458	2.460
rs2067079 (TT vs CT & CC)	1.515	0.033*	4.548	1.127	18.348
rs1625579 (TT vs GT & GG)	2.829	< 0.0001*	16.929	7.872	36.405
miR-137 serum expression	0.645	0.057	1.905	0.981	3.700
Constant	−7.701	0.000	0.000		

*GAS-5* lncRNA growth arrest-specific 5, *miR-137* microRNA 137, *CI* confidence interval.

*Significant.

## Discussion

Chronic infection with HBV is associated with severe clinical consequences including HCC development ^17^. We have explored the influence of the expression levels of GAS5 and its rs2067079 SNP and miR-137 and its rs1625579 SNP on CHBV susceptibility, and we have demonstrated them as potential genetic biomarkers.

In our study, GAS5 expression was significantly down-regulated in CHBV patients compared to controls. Our results were consistent with Feng et al., who reported down-regulation of GAS5 in patients with CHBV when compared to healthy controls^18^. Furthermore, in a study that identified the lncRNAs expression patterns in CHBV, GAS5 was down-regulated as compared to the healthy controls. However, our results were inconsistent with previous studies performed by Tu et al*.* and Chang et al., who determined that there was no significant correlation between GAS5 expression and HBV infection in patients with HCC^19,20^.

Accumulating evidence reveals that dysregulated GAS5 has been documented in many human diseases including malignancy^21^, childhood pneumonia^22^, autoimmune disorders^23^, heart failure, diabetes mellitus^24^, and neuropsychiatric disorders^24^. Importantly, GAS5 is widely considered as a tumor suppressor. It has been found to suppress liver fibrosis and inhibit the migration and invasiveness of HCC cells through miR-21 sequestration affecting cell survival^25^.

It is worth noting that both IFN‐α and IFN‐λ levels remained low during HCV infection after GAS5 overexpression, revealing the ability of GAS5 to inhibit innate immune responses after viral infection^18^.

In this study we detected, for the first time up to our knowledge, a significant down-regulation in serum miR-137 expression level in CHBV patients compared to controls. Also, it was significantly high with very low HBV viral load (less than 20 copies/ml) in contrast to those with high viral load (> 10^4^ copies/ml) among CHBV cases (*p* = 0.035). It was found that HBV X protein (HBx), a protein produced by HBV, can modulate the expression and activity of many genes and epigenetic molecules like lncRNAs and miRNAs resulting in the dysfunction of several pathways^3^. Also, cellular miRNAs can regulate HBV infection by targeting transcription factors or by direct binding to HBV transcripts.

Gao et al*.* found that HBx can down-regulate miR-137 expression by stimulating miR-137 methylation in the HCC cell line, MHCC97H. They also reported that overexpression of miR-137 suppressed HCC cell proliferation in HBx-treated MHCC97H cells by targeting Notch1^26^. Furthermore, HBx knockdown decreased the methylation of miR-137 and reconditioned the expression of miR-137^27^.

Interestingly, miR-137 overexpression decreased the cancer-initiating cell features, impaired invasion abilities, and metastasis-associated properties of HCC cells^26^. Depending on the observations of many studies, miRNAs expression is stable, unlike antigens, because they are not directly dependent on the body’s immune response. Thus, miRNAs are considered excellent biomarkers associated with HBV-related HCC^28^. It was reported that miR-137 was down-regulated in HCC cells, suggesting a potential therapeutic role of miR-137 in HCC treatment^29^.

Our results demonstrated that both GAS5 and miR-137 serum expression levels had a significant positive correlation with each other. These results were in agreement with Bian et al., who reported that there was a positive association between GAS5 and miR-137 in melanoma tissues and GAS5 was positively regulated the expression of miR-137. Simultaneously, it was determined that GAS5 could inhibit cell proliferation, migration, and invasion of melanoma cells through miR-137^30^. However, a previous study indicated that the expression of miR-137 was negatively associated with GAS5 up-regulation in the mice exposed to occlusion of the middle cerebral artery and to oxygen–glucose deprivation that stimulated primary brain neurons^31^.

Regarding GAS5 rs2067079 (C/T) in our study, the mutant CT and TT genotypes represented an increased risk of CHBV while the normal CC genotype was defined as a protective factor against CHBV risk. Up to our knowledge, no one has linked GAS5 rs2067079 with CHBV risk or pathogenesis although differentially expressed levels of GAS5 have been documented in many studies. It was reported that GAS5 rs2067079 (TT vs CC) was associated with chemo-radiotherapy induced severe myelosuppression and severe neutropenia in patients with nasopharyngeal carcinoma^32^. It was also found that this SNP was associated with the risk of development of bladder cancer^33^, multiple sclerosis^23^, and systemic lupus erythematosus (SLE)^34^.

Besides, our findings showed that the serum GAS5 expression level was significantly higher in the risky rs2067079 TT genotype compared to the CT or CC genotypes in CHBV patients. The supporting ChIP-seq data from different human cell types revealed that the site of the rs2067079 site was considered as a strong promoter region. Since the expression levels could be regulated by genetic variants in regulatory elements, this SNP might affect GAS5 transcriptional activity. It was revealed that rs2067079 has a strong feature of expression in many tissues. Furthermore, rs2067079 had a strong effect on GAS5 secondary structure which is critical for its performance^32^. Inconsistent with our results, Li et al., 2017 determined that the rs2067079 did not affect GAS5 expression levels among Chinese Han patients with SLE^34^. This disagreement may be due to the differences in ethnicity and disease mechanisms.

Furthermore, regarding miR-137 rs1625579, the mutant TT genotype and the T allele were predominantly found in CHBV cases as compared to controls. Also, there was a strong association between rs1625579 and CHBV risk. The mutant genotype TT was detected as a significant risk factor while the normal GG genotype was reported as a protective factor for CHBV. These results are suggesting a potential role of this SNP in CHBV pathogenesis.

Up to our knowledge, we demonstrated for the first time the influence of miR-137 polymorphism on CHBV susceptibility. MiR-137 rs1625579 was investigated mainly in neurodegenerative diseases such as schizophrenia. In a genome-wide association study, the strong association of the miR-137 locus with schizophrenia was evidenced^35^. Furthermore, the rs1625579 polymorphism, which is an intron SNP in the miR-137 gene, was associated with the onset of schizophrenia in Asian and European populations^36^.

Regarding the rs1625579, we reported a significant down-regulation in serum miR-137 expression level with the mutant TT genotype against GT and GG genotypes among CHBV patients. It was demonstrated that genetic variation of the miR-137 may affect the transcription process or binding ability between the miRNA and its target genes and contribute to its abnormal expression, eventually triggering the onset of many disorders^37^. It was indicated that variations in the non-coding region of miR-137 locus lead to defective mRNA structure, splicing, and stability^38^. Decreased miR-137 expression was associated with rs1625579 in different neuropsychiatric disorders^39^. Conversely, in another genotype-tissue expression study, the miR-137 locus was proved to correlate with increased miR-137 expression, especially with rs1702294, particularly, in the hippocampus of carriers of the schizophrenia-associated genotype^40^.

MiRNAs, lncRNAs, and their target genes are emerging as biomarkers of many diseases^18^. Our study is the first to investigate miR-137 expression level in CHBV patients which could be considered as a probable diagnostic marker for these patients. The role of GAS5 and miR-137 and their functioning SNPs as potential genetic predictors for CHBV risk was reported in this study. Thus interestingly, we demonstrated the associated effect of genetic and epigenetic regulatory mechanisms controlling CHBV pathogenesis. Also, the development of new therapeutic strategies based on non-coding RNAs may be a good alternative to the current conventional therapy for HBV as the conventional treatment options only does not cure it but only suppress the replication of the virus. Therefore, a lifelong treatment is essential^41^. Additionally, Peg-IFN-α has a low response rate and difficult to tolerate^42^. The current conventional therapy can reduce but not eliminate the risk for HCC^42^.

Considering our observations in CHBV, more studies with larger sample size are essential to document our findings. Although population homogeneity reduces genetic variability; our reports should be considered in large independent population studies. Further studies are needed to evaluate the exact role of the rs2067079 and rs1625579 SNPs on the tissue level of GAS5 and miR-137, respectively.

In conclusion, we identified that the expression levels of GAS5 and miR-137 were down-regulated significantly among CHBV patients. Our findings indicated that rs2067079 within GAS5 and rs1625579 within miR-137 were contributed to the pathogenesis of CHBV and could serve as potential genetic biomarkers for CHBV susceptibility. We found that the CC genotype of rs2067079 (C/T) was a protective factor while the CT and TT genotypes showed an increased risk of CHBV. Regarding rs1625579 (G/T), it was demonstrated that the normal GG genotype was a protective factor while the mutant genotype TT was reported as a risk factor for CHBV. These potential genetic biomarkers could provide a better understanding of the pathogenesis of CHBV and this allows for the development of possible therapeutic approaches.

## Subjects and methods

### Subjects

The present case–control research was conducted on 117 patients with chronic HBV infection as well as 120 control individuals who had previous HBV infection, attending the outpatient clinic, at the Department of Internal Medicine, Fayoum University hospital, Fayoum, Egypt.

The diagnosis of CHBV was based on the presence of hepatitis B virus DNA (either quantitative, or qualitative,) or detection of HBsAg (hepatitis B surface antigen) or HBeAg (hepatitis B e-antigen), in addition to the absence of anti-HBc IgM (immunoglobulin M antibodies to HBV core antigen)^43^. Any clinical and/or fibroscan (transient elastography) findings compatible with chronic liver disease were also considered.

Detailed personal and medical history was taken from each subject. Also, routine laboratory investigations such as tests for assessment of liver function, renal function tests as well as complete blood count (CBC) were determined.

The selected control individuals had positive anti-HBs IgG and anti‐HBc IgG without detection of HBsAg in addition to normal liver function tests.

The exclusion criteria for all participants comprised: (1) evidence of other types of hepatic disorders (2) antiviral treatments or immunosuppressant drugs (3) presence of positive anti-HCV or anti-HIV antibodies (4) smoking more than one cigarette pack per day.

The Ethics Committee of the Fayoum university hospital has approved this research under the Declaration of Helsinki. Each participant has given written informed consent.

### Handling blood samples

Venous blood samples which were withdrawn from each subject in the current work were deposited into plain vacutainers in which a gel separator was included. The samples were left to clot for 15 min and centrifuged at 4000×*g* for 10 min allowing the serum to be separated. Enzyme-linked immunosorbent assay (ELISA) was done on serum samples to measure HBeAg (Sunlong Biotech, China), anti‐HBc, and HBsAg (Bioelisa, Biokit, Barcelona, Spain).in agreement with the manufacturer's instruction. HBV DNA was measured using COBAS ampliprep/COBAS TAQMAN HBV TEST V2.0 kit (Roche Diagnostics) following the steps recommended by the manufacturer.

The remaining serum samples were directly stored at −80 ˚C till the time of RNA extraction. EDTA-containing tubes were used to collect additional whole blood samples to be used in DNA extraction and genotyping of rs2067079 and rs1625579.

### Extraction of total RNA and reverse transcription

Total RNAs (including microRNAs and lncRNAs) were extracted from the abovementioned serum samples by using a miRNeasy extraction kit (Qiagen, Hilden, Germany) after the addition of QIAzol lysis reagent following the manufacturer's protocol. NanoDrop (ND)-1000 Spectrophotometer (NanoDrop Technologies, Inc. Wilmington, USA) was used to determine the purity of the extracted RNA.

Reverse-transcription of the extracted RNAs was performed using RT2 first strand kit (Qiagen, Maryland, USA) with a whole volume of 20 μl/ reaction for GAS5 testing. MiR-137 was analyzed by miScript II RT Kit (Qiagen Maryland, USA) using a 20 μl RT reaction mix following manufacturers’ instructions.

### GAS5 and miR-137 quantitation by RT-qPCR

Assessment of GAS5 was evaluated by the use of RT2 SYBR Green PCR kit (Qiagen, Maryland, USA). However, the miScript SYBR Green PCR kit (Qiagen, Valenica, CA, USA) was used in the assessment of miR-137 according to the kit instructions with the aid of Rotor gene Q System (Qiagen) on 20 μl reaction mixture.

The RefSeq Accession no. of GAS5 was NR_002578.2 and the catalog number of miR-137 was MS00003486. Assessment of GAS5 was done per the following cycling steps: 95 °C for 10 min, afterward, 40 cycles at 95 °C for 15 s and 60 °C for 60 s. On the other hand, assessment of miR-137 was performed according to the subsequent cycling steps: 95 °C for 30 min, subsequently, 40 cycles at 94 °C for 15 s, 55 °C for 30 s, and 70 °C for 30 s.

GAS5 expression values were normalized by the use of GAPDH as an internal control^44,45^**.** Though, miR-137 expression values were normalized using SNORD 68 as an internal reference gene. The GAPDH primer sequences were forward, 5′-CCCTTCATTGACCTCAACTA-3′, and reverse 5′-TGGAAGATGGTGATGGGATT-3′. The catalog number of SNORD 68 was MS00033712. Calculation of the fold change (FC) of GAS5 and miR-137 was done with the aid of the equation 2^−ΔΔCt^^46^**.** The FC of control individuals was assumed as 1.

### Genotyping of rs2067079 and rs1625579

According to the instructions of the kits, genomic DNA was extracted from whole blood through a Qia-amplification DNA extraction kit (Qiagen, USA). To estimate the purity and quantity of DNA samples, NanoDrop (ND)-1000 spectrophotometer (NanoDrop Technologies, Inc. Wilmington, USA) was used. Predesigned TaqMan SNP genotyping assays (Applied Biosystems, Thermo Fisher Scientific, USA) were used to genotype both rs2067079 C/T [Assay ID: C_16166809_20] and rs1625579 G/T [Assay ID: C_8946584_20] following manufacturer’s instructions. Rotor gene Q System (Qiagen) was used in RT-PCR analyses. Cycling steps were along these lines: denaturation for 10 min at 95 °C, 45 cycles for 15 s at 92 °C, and 60 °C for annealing and extension at 90 s.

### Statistical methods

The collected data were organized and statistically analyzed using SPSS software statistical computer package version 16 (SPSS, Inc., USA). For quantitative data, the mean, median, standard deviation, and range were calculated. The Kolmogorov–Smirnov test was performed as a test of normality. The Mann–Whitney U test or Kruskal–Wallis test was used to compare any 2 groups or 3 groups, respectively. Qualitative data were presented as numbers and percentages, and chi-square (× 2) was used as a test of significance. Spearman's correlation was used to test the association of quantitative variables. ROC curve was done to evaluate the diagnostic performance of the biomarkers and the best cut-off point of the biomarkers was determined, Odds ratio (OR) with 95% confidence was calculated for different forms of polymorphism to identify its association with disease. Multiple logistic regression analysis was done to detect the most significantly associated factors with CHBV disease. The homozygote genotype or the major allele detected among the control subjects was selected as the reference group. Significance was defined as *p* < 0.05.
